# Ketone-selenoesters as potential anticancer and multidrug resistance modulation agents in 2D and 3D ovarian and breast cancer in vitro models

**DOI:** 10.1038/s41598-022-10311-y

**Published:** 2022-04-21

**Authors:** Simona Dobiasová, Nikoletta Szemerédi, Denisa Kučerová, Kamila Koucká, Radka Václavíková, Helena Gbelcová, Tomáš Ruml, Enrique Domínguez-Álvarez, Gabriella Spengler, Jitka Viktorová

**Affiliations:** 1 Department of Biochemistry and Microbiology, Faculty of Food and Biochemical Technology, University of Chemistry and Technology Prague, Technická 3, 166 28 Prague 6, Czechia; 2 Department of Medical Microbiology, Albert Szent-Györgyi Medical School, University of Szeged, Semmelweis utca 6, Szeged, 6725 Hungary; 3 Toxicogenomics Unit, National Institute of Public Health, Šrobárova 49, 100 00 Prague, Czechia; 4 Laboratory of Pharmacogenomics, Biomedical Center, Faculty of Medicine in Pilsen, Charles University, Alej Svobody 1655, 323 00 Pilsen, Czechia; 5 Institute of Medical Biology, Genetics and Clinical Genetics, Faculty of Medicine, Comenius University in Bratislava, Sasinkova 4, 811 08 Bratislava, Slovakia; 6 Instituto de Química Orgánica General (IQOG-CSIC), Consejo Superior de Investigaciones Científicas, Juan de la Cierva 3, 28006 Madrid, Spain

**Keywords:** Cancer, Molecular medicine

## Abstract

Long-term treatment of cancer with chemotherapeutics leads to the development of resistant forms that reduce treatment options. The main associated mechanism is the overexpression of transport proteins, particularly P-glycoprotein (P-gp, ABCB1). In this study, we have tested the anticancer and multidrug resistance (MDR) modulation activity of 15 selenocompounds. Out of the tested compounds, **K3**, **K4**, and **K7** achieved the highest sensitization rate in ovarian carcinoma cells (HOC/ADR) that are resistant to the action of the Adriamycin. These compounds induced oxidation stress, inhibited P-gp transport activity and altered ABC gene expression. To verify the effect of compounds, 3D cell models were used to better mimic in vivo conditions. **K4** and **K7** triggered the most significant ROS release. All selected selenoesters inhibited P-gp efflux in a dose-dependent manner while simultaneously altering the expression of the ABC genes, especially P-gp in paclitaxel-resistant breast carcinoma cells (MCF-7/PAX). **K4**, and **K7** demonstrated sensitization potential in resistant ovarian spheroids. Additionally, all selected selenoesters achieved a high cytotoxic effect in 3D breast and ovarian models, which was comparable to that in 2D cultures. **K7** was the only non-competitive P-gp inhibitor, and therefore appears to have considerable potential for the treatment of drug-resistant cancer.

## Introduction

Cancer is the second most common global cause of death behind cardiovascular disease. In 2020, Global Cancer Observatory (GCO) reported that nearly 2.3 million women were diagnosed with breast cancer (BC) and around 314,000 with ovarian cancer (OC)^1^. During the treatment process, some tumours activate specific cellular mechanisms by which their cells acquire the ability to develop resistances to multiple drugs, what is known as multidrug resistance (MDR)^2^. The pathogenesis of MDR in cancer is highly complex and involves several factors. The main mechanism associated with MDR development appears to be overexpression of genes encoding the transport proteins (ABC) affecting the concentrations of chemotherapeutics in tumours^3^.

ATP-binding cassettes (ABCs) represent a superfamily of efflux proteins that utilize the energy from ATP hydrolysis to transport specific molecules. So far, 48 human ABC transporters, grouped into 7 subfamilies (ABCA–ABCG), form the human ABC superfamily^4^. Their primary function is associated with the translocation of several substrates across the plasma membrane, including glycolipids, phospholipids, steroids, and xenobiotics. In healthy cells, the pumps positively influence drug ADME properties (Absorption, Distribution, Metabolism, and Excretion), membrane homeostasis, cell signalling, and detoxification. Due to their widespread functions, the upregulation of ABC-encoding genes is related to the development of pathologies such as neurodegenerative diseases (e.g., Alzheimer’s and epilepsy) and cancer progression^5,6^. Out of the ABCs, P-glycoprotein (P-gp) appears to be the efflux pump most involved in tumour chemoresistance^7^.

P-gp (ABCB1), also known as multidrug resistance protein 1 (MDR1), was the first ABC transporter discovered. It was firstly reported in Chinese hamster ovary cells about 40 years ago^8^. Due to the expression in various tissues, P-gp limits drug penetration after oral administration (expression in the luminal membrane of enterocytes) and secretes drug into bile and urine (expression in hepatocyte membrane). As a component of sensitive tissues (e.g., in blood–brain barrier (BBB), lymphocytes and endothelial cells in foetal vessels), it affects drug penetration and eliminates negative effects of toxic substances^9^. P-gp has a broad structural and functional substrate specificity for compounds such as steroid hormones, immunosuppressants, antibiotics, and anticancer agents (e.g., vinca alkaloids, anthracyclines, epipodophyllotoxins)^10^. Other ABC proteins associated with the MDR phenotype—MRP1 (ABCC1, multidrug resistance-associated protein 1), and BCRP (ABCG2, breast cancer protein) are involved in the detoxification of hydrophobic organic molecules and xenobiotics^11,12^. Many of the chemotherapeutics (e.g., Adriamycin, mitoxantrone, and etoposide) belong to the substrates for all three transmembrane pumps. Due to long-term treatment with these antitumour agents, chemoresistance is not only common, but also expected. For instance, 90% of primary breast cancers are sensitive to systemic agents in contrast to only 50% of metastases^13^. Therefore, new strategies to overcome this problem are more than desirable.

A substantial group of MDR modulators represents the selenium-containing compounds. In general, selenocompounds are divided into two main categories: inorganic (e.g., selenium dioxide, selenites) and organic (e.g., selenides, seleninic acids, selenoesters, selol, seleno-amino acids, and other)^14^. These compounds affect the cancer progression by activating cell death, oxidation stress, and the immune response^15–17^. In addition to anticancer potential, they cooperate synergistically with specific cytostatics, and thus they enhance chemotherapeutic efficiency in drug-resistant tumours. For example, the combination of sodium selenite and docetaxel increased growth inhibition of prostate metastatic cancer line by 67%, compared to sodium selenite (24%) and docetaxel (22%) alone^15^. Furthermore, selenoesters with ketone terminal fragment inhibited P-gp in drug resistant T-lymphocytes more than verapamil (competitive P-gp inhibitor)^16^. However, the search for new interesting Se-compounds with promising biological activity is still ongoing. There are not many studies of Se-compounds dealing with MDR in its complexity from a direct inhibition of P-gp modulation to the expression of genes encoding ABC proteins. In addition, researchers usually focus on testing the inhibitors in 2D cell cultures instead of more relevant 3D spheroid models.

**Figure 1:**
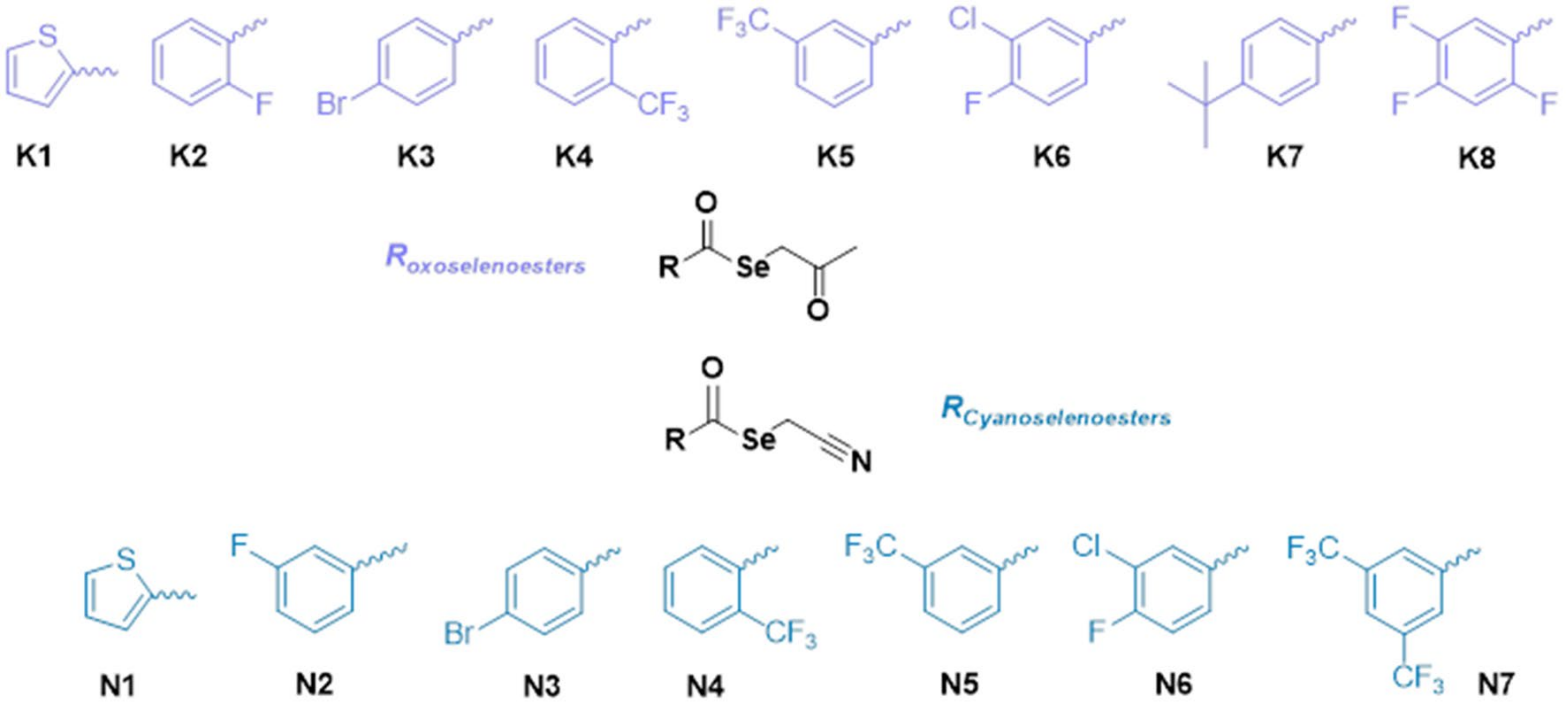
The structures of 15 selenocompounds used in this work: **K1–K8** ketone-selenoesters and **N1–N7** cyanoselenoesters.

To address this issue, we investigated the anticancer and MDR modulation activity of 15 (Fig. 1) selenocompounds. Regarding the sensitization of drug-resistant ovarian and breast adenocarcinomas (HOC/ADR, MCF-7/PAX), three ketone-selenoesters **K3**, **K4**, **K7** were analysed for the induction of oxidation stress. In addition, we demonstrated the ability of these compounds to modulate MDR by direct inhibition of P-gp and downregulation of genes encoding ABC proteins associated with resistance. To better mimic in vivo conditions, we observed the effect of selected selenoesters on formation of 3D ovarian and breast cultures. Additionally, the cytotoxic and MDR modulation activity of selected compounds was evaluated in both spheroid models. Our results confirmed that some of the tested ketone-selenoesters are potential anticancer and drug-resistance reversing agents.

## Results

### Cytotoxic activity of selenoesters against several cancer and a non-cancerous cell line

Cytotoxic potential of selenocompounds was determined in several cancer cell lines (sensitive and resistant sub-lines) and in a non-cancerous cell line (Tables 1, 2). To monitor their toxic activity specifically in cancer cells/resistant cancer cells, the selectivity/collateral sensitivity indexes were calculated. In the case of the HOC and its sub-line HOC/ADR, the ketone-selenoesters demonstrated a higher cytotoxic potential against sensitive cells compared to the resistant ones; the collateral sensitivity was not observed (Table 1). The ketone selenoesters **K1**, **K2**, and **K6** showed the most significant decrease in cell viability (HOC) with IC_50_ less than 1 µM. Cyano-selenoesters showed a lower decrease in cell viability, resulting in a higher IC_50_ in both HOC and HOC/ADR in comparison to ketone selenocompounds. Compound **N2** and **N3** demonstrated slight collateral sensitivity against HOC/ADR.

**Table 1:** Cytotoxic potential of selenocompounds against sensitive ovarian (HOC) and breast carcinoma (MCF-7) cell lines and their Adriamycin (ADR) and paclitaxel (PAX) resistant sub-lines with the corresponding collateral sensitivity (CS) index.

Cpd	HOC	HOC/ADR	CS	MCF-7	MCF-7/PAX	CS
	IC_50_ [µM]			IC_50_ [µM]		
K1	0.8 ± 0.0^b^	2.3 ± 0.0^a^	0.4 ± 0.0	1.6 ± 0.1^b^	2.2 ± 0.2^b,c,d,e^	0.7 ± 0.1
K2	0.8 ± 0.1^b^	2.4 ± 0.1^a^	0.3 ± 0.0	1.9 ± 0.1^b,c,d^	1.9 ± 0.1^b,c^	1.0 ± 0.1
K3	1.8 ± 0.0^c^	2.6 ± 0.1^a^	0.7 ± 0.1	2.0 ± 0.5^b,c,d,e^	2.1 ± 0.1^b,c,d^	1.0 ± 0.3
K4	2.2 ± 0.1^c,d^	3.6 ± 0.1^b^	0.6 ± 0.0	1.8 ± 0.1^b,c,d^	3.3 ± 0.3^h^	0.6 ± 0.0
K5	1.1 ± 0.0^b^	2.2 ± 0.3^a^	0.5 ± 0.1	1.6 ± 0.1^b,c^	2.8 ± 0.1^g^	0.6 ± 0.1
K6	0.7 ± 0.1^b^	2.6 ± 0.1^a^	0.3 ± 0.0	2.1 ± 0.2^b,c,d,e^	2.3 ± 0.3^c,d,e^	0.9 ± 0.1
K7	1.1 ± 0.1^b^	3.1 ± 0.3^a,b^	0.4 ± 0.1	1.9 ± 0.1^b,c,d^	2.4 ± 0.1^d,e,f^	0.8 ± 0.1
K8	2.1 ± 0.1^c,d^	2.7 ± 0.0^a^	0.8 ± 0.1	2.6 ± 0.4^e,f^	2.0 ± 0.1^b,c^	**1.3 ± 0.3**
N1	2.7 ± 0.3^e,f^	4.2 ± 0.2^d^	0.7 ± 0.1	3.1 ± 0.2^f^	2.5 ± 0.0^e,f,g^	**1.2 ± 0.2**
N2	7.1 ± 0.2^h^	4.7 ± 0.1^c^	**1.5 ± 0.1**	2.6 ± 0.1^e,f^	2.5 ± 0.3^e,f,g^	1.0 ± 0.1
N3	6.5 ± 0.3^g^	5.2 ± 0.1^c,d^	**1.3 ± 0.1**	2.3 ± 0.5^c,d,e^	2.7 ± 0.2^f,g^	0.9 ± 0.3
N4	2.4 ± 0.1^d,e^	3.7 ± 0.2^b^	0.6 ± 0.1	2.1 ± 0.1^b,c,d,e^	2.4 ± 0.2^d,e,f^	0.9 ± 0.1
N5	2.3 ± 0.1^d^	4.5 ± 0.2^c^	0.5 ± 0.1	2.5 ± 0.2^d,e,f^	2.7 ± 0.2^f,g^	0.9 ± 0.2
N6	2.4 ± 0.2^d,e^	3.6 ± 0.0^b^	0.7 ± 0.1	1.7 ± 0.0^b,c^	1.9 ± 0.1^b^	0.9 ± 0.0
N7	2.9 ± 0.1^f^	5.7 ± 0.2^d^	0.7 ± 0.0	2.2 ± 0.1^b,c,d,e^	2.7 ± 0.3^f,g^	0.8 ± 0.1
ADR	0.01 ± 0.0^a^	2.5 ± 0.0^a^	0.0 ± 0.0	–	–	–
PAX	–	–	–	0.03 ± 0.0^a^	1.5 ± 0.0^a^	0.0 ± 0.0

Data are expressed as the average inhibitory concentration (IC_50_) and collateral sensitivity index (CS) of three repetitions with standard error of the mean (SEM). The selectivity is considered as a strongly selective, if CS value is higher than 6, moderately selective if 6 > CS > 3, slightly selective if 3 > CS > 1 and non-selective if CS is less than 1. The statistical differences between compounds were calculated by one-way analysis of variance (ANOVA) and Duncan’s post hoc test (p ˂ 0.05) within one cell line, where different letters (a-h) denoted the significances; the different cell lines were evaluated independently on each other.

Significant values are in [bold].

**Table 2:** Cytotoxic potential of selenoesters against non-cancerous cell line HEK293 with the corresponding therapeutic index.

Cpd	HEK293	SI			
	IC_50_ [µM]	HEK293/HOC	HEK293/HOC/ADR	HEK293/MCF-7	HEK293/MC7-7/PAX
K1	1.0 ± 0.1^a^	1.2 ± 0.1	0.4 ± 0.0	0.6 ± 0.1	0.4 ± 0.0
K2	1.2 ± 0.1^a,b^	1.6 ± 0.1	0.5 ± 0.0	0.7 ± 0.1	0.6 ± 0.1
K3	2.4 ± 0.0^c^	1.3 ± 0.0	0.9 ± 0.0	1.2 ± 0.3	1.1 ± 0.1
K4	5.4 ± 0.4^d,e,f^	2.5 ± 0.2	1.5 ± 0.2	2.9 ± 0.3	1.6 ± 0.2
K5	1.6 ± 0.0^a,b,c^	1.5 ± 0.0	0.7 ± 0.1	1.0 ± 0.1	0.6 ± 0.0
K6	2.0 ± 0.1^b,c^	2.7 ± 0.4	0.8 ± 0.1	0.9 ± 0.2	0.9 ± 0.1
K7	1.5 ± 0.1^a,b^	1.7 ± 0.2	0.5 ± 0.1	0.8 ± 0.1	0.6 ± 0.1
K8	2.0 ± 0.2^b,c^	0.9 ± 0.1	0.7 ± 0.0	0.8 ± 0.1	1.0 ± 0.2
N1	6.0 ± 0.6^e,f,g^	2.2 ± 0.4	1.4 ± 0.2	2.0 ± 0.3	2.4 ± 0.4
N2	7.8 ± 0.2^h^	1.1 ± 0.1	1.7 ± 0.1	**3.0 ± 0.2**	**3.1 ± 0.2**
N3	8.0 ± 0.3^h^	1.2 ± 0.1	1.5 ± 0.1	**3.5 ± 0.9**	**3.0 ± 0.6**
N4	5.2 ± 0.3^d,e^	2.2 ± 0.2	1.4 ± 0.1	2.5 ± 0.2	2.1 ± 0.2
N5	6.0 ± 0.2^f,g^	2.7 ± 0.2	1.3 ± 0.1	2.4 ± 0.3	2.3 ± 0.2
N6	4.9 ± 0.1^d^	2.1 ± 0.2	1.4 ± 0.0	2.9 ± 0.1	2.6 ± 0.1
N7	6.6 ± 0.6^f^	2.3 ± 0.3	1.2 ± 0.1	**3.0 ± 0.5**	2.5 ± 0.4

Data are expressed as the average inhibitory concentration (IC_50_) and selectivity index (SI) of three repetitions with standard error of the mean (SEM). The selectivity is considered as strongly selective, when SI value is higher than 6, moderately selective when 6 > SI > 3, slightly selective when 3 > SI > 1 and non-selective if SI is less than 1. The statistical differences between compounds were calculated by one-way analysis of variance (ANOVA) and Duncan’s post hoc test (p ˂ 0.05), where the significances were denoted by different letters (a–h).

Significant values are in [bold].

The capacity of the tested selenocompounds to inhibit cell growth was determined on non-cancerous embryonic kidney cell line (HEK293, Table 2). One ketone-(**K4**) and all the cyanoselenoesters (**N1–7**) showed lower cytotoxic effect against HEK293 with the IC_50_ in the concentration range from 4.9 to 8.0 µM than ketone-selenoesters, which caused a strong decrease of cell viability with IC_50_ from 1 to 2.4 µM. In correlation with cancer cell lines, the most significant toxicity was shown by **K1–2**, **K5**, **K7**. Regarding cyanoselenoesters, the selectivity indexes calculated between HEK293 and cancer cell lines from 1.1 to 3.5, thus these compounds demonstrated slight or moderate (**N2–3**, **N7**) selectivity against cancer cells. Among all ketone-selenoesters, **K4** showed the highest selectivity against all cancer cell lines tested herein.

The IC_50_ values of selenoesters were quite consistent in both the MCF-7 and its sub-line MCF-7/PAX. The most significant cytotoxic effect against both cell lines was obtained by **K1**–**7**, **N4** and **N6**–**N7**. The selectivity indexes in both ovarian and breast cell lines were mostly < 1. Compounds **K8** and **N1** demonstrated slight collateral sensitivity against paclitaxel-resistant breast carcinoma.

### Sensitization of MDR cell lines by selenoesters

To determine the sensitization potential of compounds, HOC/ADR and MCF-7/PAX were used. Based on the cytotoxic potential of the compounds against several cancer and non-cancerous cell lines (Table 1), the concentrations of applied compounds were adjusted. IC_10_ values was considered as the concentrations, which should not decrease the cell viability under 90%. After the addition of compounds, Adriamycin (concentration range 0.1–20 µM) and paclitaxel (concentration range 0.1–10 µM) were added to HOC/ADR and MCF-7/PAX, respectively. The sensitization potential of the compounds was determined as the ratio of the IC_50_ of cytostatic agent (Adriamycin/paclitaxel) to the IC_50_ of cytostatic agent affected by the compound in concentration corresponding to the IC_10_ value. Fold change greater than 1 indicated synergic effect of cytostatic agent and the tested compound.

The most significant result was shown by compound **K7** for the Adriamycin-resistant cell line (HOC/ADR) (Table 3). The addition of **K7** (IC_10_ = 1.8 µM) decreased the IC_50_ of Adriamycin almost 6 times. A mild effect was provided by compounds **K1–K4**, **K6**, **K8**, **N2**, **N5** and **N7**. On the contrary, the addition of **K5**, **N1**, and **N6** caused decrease of the cell sensitivity against Adriamycin. Therefore, higher concentration of the cytostatic agent was needed for the cell growth inhibition. **K5** increased the IC_50_ of Adriamycin almost twofold, **N1** 3.5 fold and **N6** almost threefold.

**Table 3:** Sensitization potential of selenoesters against the Adriamycin-resistant human ovarian carcinoma cell line HOC/ADR. Tested compounds were applied in single dose (IC_10_) and Adriamycin in the indicated concentration range (0.1–20 µM) in order to determine its concentration halving the cell viability (IC_50_). Fold change was calculated as the ratio of IC_50_ of Adriamycin and IC_50_ of Adriamycin in the presence of IC_10_ dose of compounds.

Cpd	IC_10_ of compounds (µM)	IC_50_ of adriamycin (µM)	Fold change
K1	1.5	1.8 ± 0.1^b^	(1.3 ± 0.1)×
K2	1.0	2.1 ± 0.0^b^	(1.2 ± 0.0)×
K3	2.0	1.7 ± 0.1^b^	(1.4 ± 0.1)×
K4	2.9	1.8 ± 0.0^b^	(1.4 ± 0.1)×
K5	1.5	4.5 ± 0.1^c^	(0.5 ± 0.1)×
K6	1.3	2.2 ± 0.1^b^	(1.1 ± 0.1)×
K7	1.8	0.4 ± 0.6^a^	**(5.9 ± 0.6)**×
K8	3.0	2.2 ± 0.0^b^	(1.1 ± 0.1)×
N1	2.5	8.7 ± 0.9^e^	(0.3 ± 0.1)×
N2	3.0	2.1 ± 0.1^b^	(1.2 ± 0.1)×
N3	3.1	2.0 ± 0.0^b^	(1.3 ± 0.0)×
N4	2.4	2.0 ± 0.1^b^	(1.3 ± 0.1)×
N5	2.5	2.1 ± 0.2^b^	(1.2 ± 0.2)×
N6	2.6	6.5 ± 0.2^d^	(0.4 ± 0.0)×
N7	3.1	2.3 ± 0.1^b^	(1.1 ± 0.1)×
ADR	–	2.5 ± 0.0^b^	–

Data are expressed as the average inhibitory concentrations (IC_50_) or fold change of three repetitions with standard error of the mean (SEM). Fold change higher than 1 indicated the synergistic effect of cytotostatic and tested compound. The statistical differences between compounds were calculated by one-way analysis of variance (ANOVA) and Duncan’s post hoc test (p ˂ 0.05), where the significances were denoted by different letters (a–e).

Significant values are in [bold].

The compounds with the highest effect on sensitization of HOC/ADR were subsequently tested on MCF-7/PAX. Like in experiments on HOC/ADR, the most significant effect was exerted by compound **K7** (Table 4). The IC_10_ dose of **K7** demonstrated twofold decrease of the paclitaxel concentration needed to halve the cell viability. However, the synergistic effect between the tested compound **K7** and cytostatic agent (twofold) was not as remarkable as with the resistant ovarian cell line (sixfold). The remaining selenoesters (**K3**, **K4**) provided comparable results on the both cell lines (HOC/ADR, MCF-7/PAX). A slight synergistic effect was observed in the case of the combination of **K3**, **K4** with paclitaxel (1.4-fold). The effect was statistically significant (p ≤ 0.0004).

**Table 4:** The sensitization potential of selenoesters against paclitaxel-resistant human breast carcinoma cell line MCF- 7/PAX. Tested compounds were applied in single dose (IC_10_) and paclitaxel in concentration range in order to determine its concentration halving the viability (IC_50_). Fold change was calculated as the ratio of IC_50_ of paclitaxel and IC_50_ of paclitaxel in the presence of IC_10_ dose of compound.

Cpd	IC_10_ of compounds [µM]	IC_50_ [µM]	Fold change
K3	1.2	1.1 ± 0.1^b^	(1.4 ± 0.1)×
K4	1.8	1.1 ± 0.0^b^	(1.4 ± 0.1)×
K7	1.3	0.7 ± 0.0^a^	**(2.1 ± 0.2)×**
PAX	–	1.5 ± 0.0^c^	–

Data are expressed as the average inhibitory concentration (IC_50_) or fold change of three repetitions with standard error of the mean (SEM). Fold change higher than 1 indicated the synergistic effect of paclitaxel and selenoesters. The statistical differences between compounds were calculated by one-way analysis of variance (ANOVA) and Duncan’s post hoc test (p ˂ 0.05), where significances were denoted by different letters (a–c).

Significant values are in [bold].

### Oxidation stress potential of selected selenoesters

Selenoesters (**K3**, **K4**, **K7**) were tested for their ability to generate reactive oxygen species (ROS). HOC was exposed to selenoesters in concentration of 1 µM for 24 h. DMSO (5% V/V) was used as a positive control. The required volume of DMSO (1% V/V) was added to untreated cells. ROS are essential biomolecules in cellular regulation as a part of the defence mechanisms. On the other hand, their participation in cell signalling may affect apoptosis and other death pathways. In the assay, dihydroethidium (DHE) reacts with superoxide anions by forming DNA-binding fluorophore ethidium bromide. Based on fluorescence, the cell population can be distinguished into ROS negative and ROS positive cells containing oxygen radicals.

All tested compounds affected ROS production to some negative extent (Table 5). In comparison to the untreated control, **K3** and **K7** increased most significantly the amount of radical positive cell population (**p < 0.01). There was no statistically significant difference between potential induced by **K3** and **K7**. **K4** triggered ROS positive events, but the effect was not as significant (*p < 0.05). DMSO (5% V/V) caused oxidation stress with massive increase of ROS positive cells.

**Table 5:** Induction of the oxidation stress by selected selenoesters in human sensitive ovarian adenocarcinoma cell line (HOC).

Cpd	Concentration (µM)	ROS(−) cells (%)	ROS(+) cells (%)
K3	1	70.8 ± 4.2**	29.1 ± 4.2**
K4	1	85.5 ± 1.6*	14.5 ± 1.5*
K7	1	77.7 ± 0.4**	22.3 ± 0.4**
Untreated cells (1% V/V)	–	92.1 ± 0.2	7.9 ± 0.2
Treated cells with DMSO (5% V/V)	–	31.8 ± 2.3***	68.7 ± 1.9***

Data are expressed as the average of the cell percentage number of three repetitions with standard error of the mean (SEM). The statistical differences between two datasets were calculated by using a two-compound t-test at the level *p < 0.05, **p < 0.01, ***p < 0.001.

### P-glycoprotein ATPase activity modulation by selected ketone-selenoesters

P-gp Glo™ Assay System represents a bioluminescent assay for the detection of P-gp ATPase activity. The reaction mixture was composed of human recombinant P-gp membrane fraction alone (without an activator/inhibitor); or with the addition of verapamil (P-gp activator), Na_3_VO_4_ (sodium orthovanadate, P-gp inhibitor) or tested compounds. The activity of P-gp is inversely proportional to the luciferase-generated luminescent signal of unmetabolized ATP. For the detection of P-gp inhibitory potential, the compounds (concentration range 12.5–100 µM) were tested for their capacity to inhibit verapamil-stimulated P-gp ATPase activity in a competitive mode. Sodium orthovanadate as a P-gp inhibitor decreased ATP consumption; residual ATP in the reaction caused a luminescent signal which was proportional to the inhibitory activity. Verapamil as P-gp activator had the opposite effect and stimulated the P-gp ATPase activity (lower luminescent signal). When the compound acted as P-gp inhibitor, the reduction of verapamil-stimulated activity was observed with higher relative luminescent signal (RLU).

**Figure 2:**
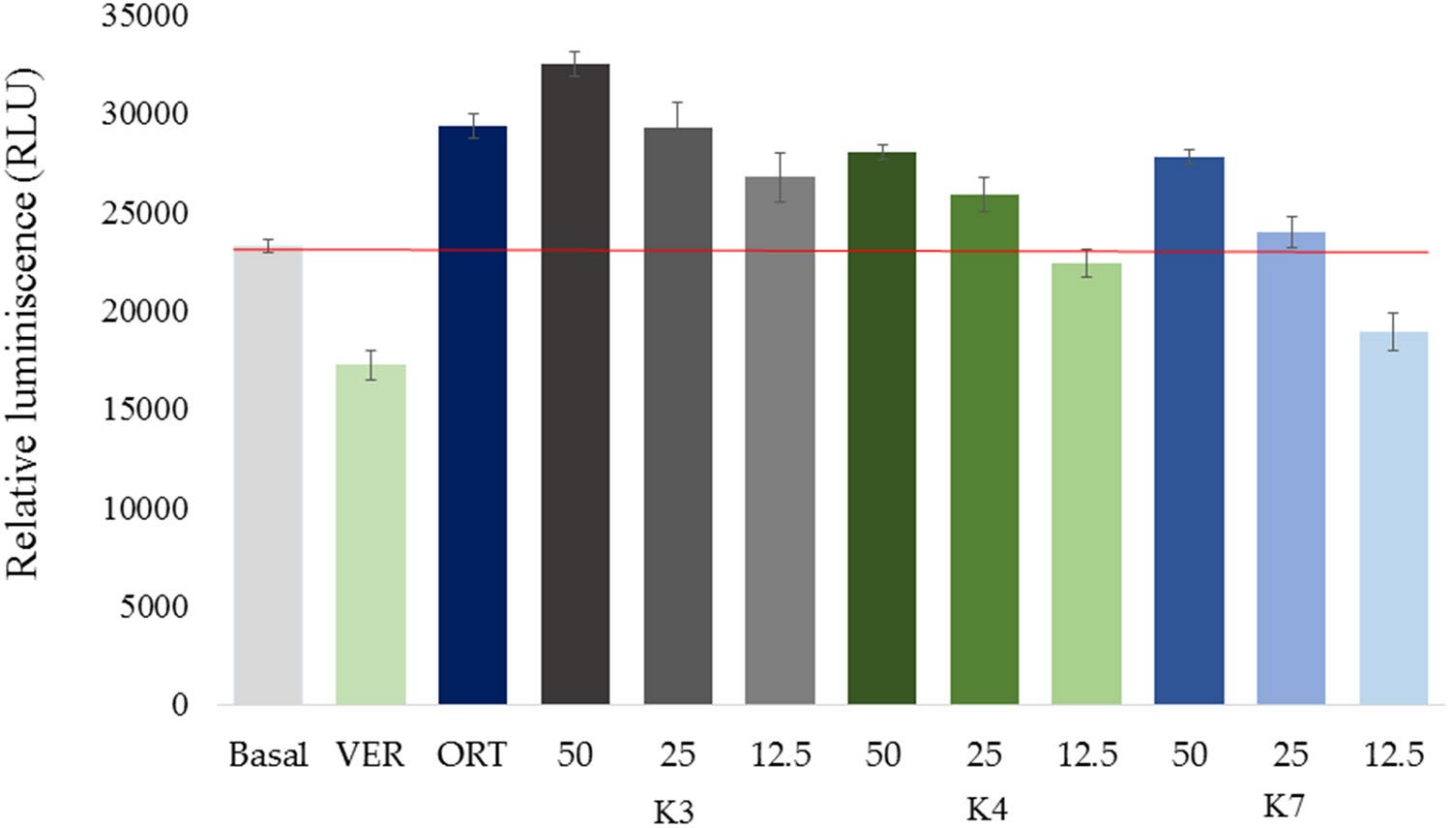
The modulation of P-glycoprotein (P-gp) ATPase activity by selected ketone-selenoesters. For the detection, P-gp kit with a human recombinant P-gp membrane fraction was used (P-gp-Glo assay system, Promega). The luminescent reaction was composed of P-gp membranes alone (without the addition of activator/inhibitor, Basal) or with the addition of verapamil (100 µM, P-gp activator), Na_3_VO_4_ (100 µM, sodium orthovanadate, P-gp inhibitor) or tested compounds. Ketone-selenoesters **K3**, **K4**, and **K7** were tested at a concentration range (12.5–100 µM) for their capacity to inhibit 100 µM verapamil-stimulated P-gp ATPase activity in a competitive mode. The red dividing line represents the interface between effect of inhibitors and activators. Data are expressed as the average of three repetitions with the standard error of the mean.

Selected ketone-selenoesters sensitizing drug-resistant cell lines were tested for their potential to directly modulate P-gp ATPase activity. The red line represents an interface between effect of inhibitors and activators (**Fig. ****2**). The level indicated by the red line demonstrates the compounds without any effect. The compounds effectively inhibiting verapamil-stimulated activity (100 µM) resulted in enhanced luminescent signal. The lowest concentration (12.5 µM) of compounds **K4** and **K7** induced RLU signals below the red line, indicating verapamil-related inhibition effect of these compounds. The most significant inhibition was observed in case of **K3** at 50 µM concentration: P-gp ATPase activity was more effectively inhibited than sodium orthovanadate (Na_3_VO_4_, 100 µM).

Using ATP standard curve and linear regression, the average amount of ATP consumed in presence of each compound was calculated. Afterwards, the specific P-gp activity (consumed ATP) was plotted versus the concentrations of the test compound and IC_50_ values were determined (Table 6). By using one-way ANOVA analysis, the statistical differences between compounds were identified; the compounds with the most statistically significant inhibition capacity was **K3**, followed by **K4** and **K7**.

**Table 6:** The modulation of the verapamil-stimulated (100 µM) P-glycoprotein ATPase activity by selected ketone-selenoesters.

Cpd	IC_50_ [µM]
K3	11.7 ± 1.0^a^
K4	23.0 ± 0.2^b^
K7	25.7 ± 0.6^c^

Data are expressed as the average inhibitory concentration (IC_50_) of three repetitions with standard error of the mean (SEM). The statistical differences between compounds were calculated by one-way analysis of variance (ANOVA) and Duncan’s post hoc test (p ˂ 0.05), where significances were denoted by different letters (a–c).

### Effect of selenoesters on expression of the genes encoding the ABC transporters

Based on the fact that compounds **K3**, **K4**, **K7** sensitized drug-resistant cell lines (HOC/ADR, MCF-7/PAX) and modulated ATPase activity of one from the ABC proteins (P-gp), we verified the potential of these compounds to affect ABC superfamily gene expression. The ovarian and breast cancer cell lines (parental cells and resistant sub-line) were analysed for the expression profile of genes encoding the ABC proteins in the previous works^18–20^. Therefore, both resistant cell lines were exposed to IC_10_ of **K3**, **K4**, and **K7** with or without the addition of IC_25_ dose of cytostatics (Adriamycin = 1.25 µM, paclitaxel = 0.75 µM). After 48 h, the effect of compounds alone or in the combination with cytostatics was observed (Supplementary Tables 1, Table 2).

The way in which the compounds acted differs significantly in various drug-resistant cell lines. In case of HOC/ADR, the tested compounds alone (**K3**, **K4**, **K7**) generally upregulated the expression of many ABC genes, especially connected with MDR—*ABCB1* (P-gp), *ABCC1* (MRP1), *ABCG2* (BRCP). On the other hand, the *ABCA3*, *ABCA7*, *ABCB2*, *ABCD1* genes were downregulated by** K3** and **K4**. **K7** also decreased the expression of *ABCA7*, *ABCB2*, *ABCD1-2*. Interestingly, all tested substances increased the expression of the whole ABCF gene subfamily (*ABCF1-3*). Despite the presence of the tested compounds, significant changes in ABC gene expression were caused only by the effector Adriamycin (Supplementary Table 1). Thus, the downregulation of the *ABCA3* gene caused by **K3**, **K4** and also the downregulation of *ABCB2* caused by **K3**, **K4**, and **K7** disappeared (Supplementary Table 1). On the other hand, the upregulation of MDR genes (*ABCB1*, *ABCC1*, *ABCG2*) was increased due to addition of a cytostatic agent.

The different impact of compounds regarding gene expression was observed on the MCF-7/PAX. All tested compounds downregulated the expression of most of the tested ABC transporter genes except for *ABCA4, ABCA12, ABCC3, ABCF1* which were upregulated (Supplementary Table 2). Additionally, all tested selenoesters decreased the expression of ABCB1 (P-gp) encoding gene, concretely **K3** by 60%, **K4** by 43%, and **K7** by 64%. In the case of other MDR genes, the tested selenoesters caused downregulation of the expression of genes encoding ABCC1 (MRP1) and ABCG2 as follows: **K3** by 73%, **K4** by 60%, **K7** by 76%, and **K3** by 75%, **K4** by 62%, **K7** by 80%, respectively. As in the previous case, the addition of the cytostatic agent (paclitaxel) reduced the compound’s impact. The most significant expression reduction was caused by the addition of **K7** (altering expression of 32 ABC genes) followed by **K4** (altering expression of 31 ABC genes) and **K3** (altering expression of 30 ABC genes). These compounds effectively downregulated the expression of MDR genes (*ABCB1, ABCC1, ABCG2*) despite the presence of paclitaxel that enhances MDR genes’ expression. Based on the observation the expression of some ABC encoding genes after combined treatment with cytostatics and **K4** versus the treatment with the **K4** only, it can be concluded, that the significant upregulation of the mentioned genes was caused by the cytostatic agent and not by **K4**. However, ABCA1, ABCB2-3, ABCC4, ABCG4 encoding genes were downregulated by each of the compounds in combination with paclitaxel.

### The impact of selenoesters on 3D spheroids formation

**Figure 3:**
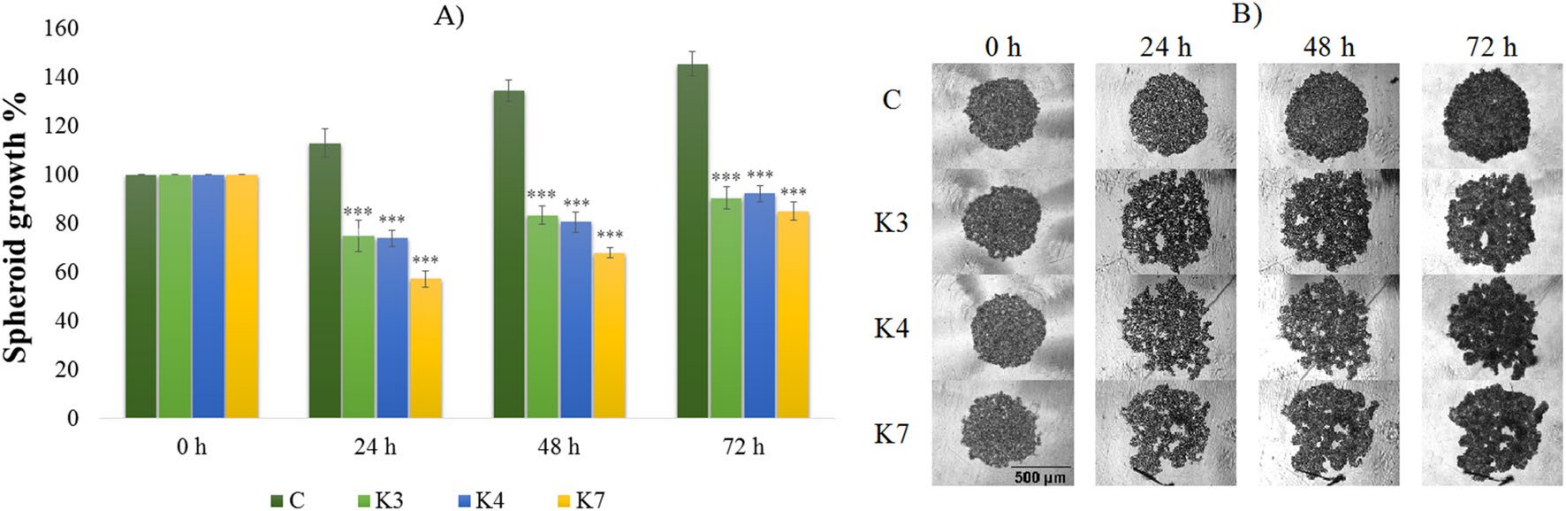
The impact of **K3**, **K4**, and **K7** (1 µM) on sensitive HOC spheroid area after 24, 48, and 72 h exposure (**A**). Representative images of sensitive HOC spheroids at 0–72 h of experiment (**B**), scale bar = 500 µm. The control spheroids (**C**) were treated with 1% V/V DMSO. After incubation, the spheroid areas were determined and the spheroid growth was calculated (**A**, relative to control). Data are expressed as the average of spheroid growth (%) of nine repetitions with the corresponding standard error of the mean. The statistical differences between the control spheroids and treated spheroids were calculated by using a two-compound t-test at the level *p < 0.05, **p < 0.01, ***p < 0.001.

**Figure 4:**
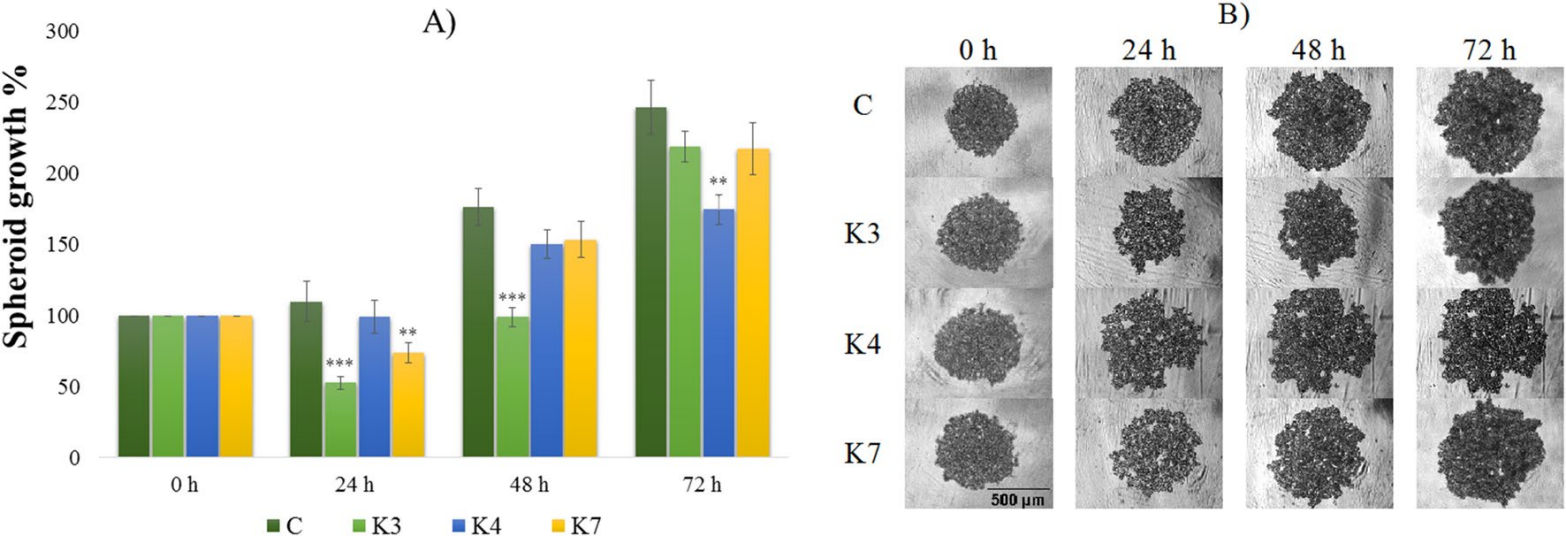
The impact of **K3**, **K4**, and **K7** (1 µM) on resistant HOC/ADR spheroids formation after 24, 48, and 72 h exposure (**A**). Representative images of resistant HOC/ADR spheroids at 0 to 72 h of experiment (**B**), scale bar = 500 µm. The control spheroids (**C**) were treated with 1% V/V DMSO. After incubation, the spheroid areas were determined and the spheroid growth was calculated (**A**, relative to control). Data are expressed as the average of spheroid growth (%) of nine repetitions with the corresponding standard error of the mean. The statistical differences between the control spheroids and treated spheroids were calculated by using a two-compound t-test at the level *p < 0.05, **p < 0.01, ***p < 0.001.

**Figure 5:**
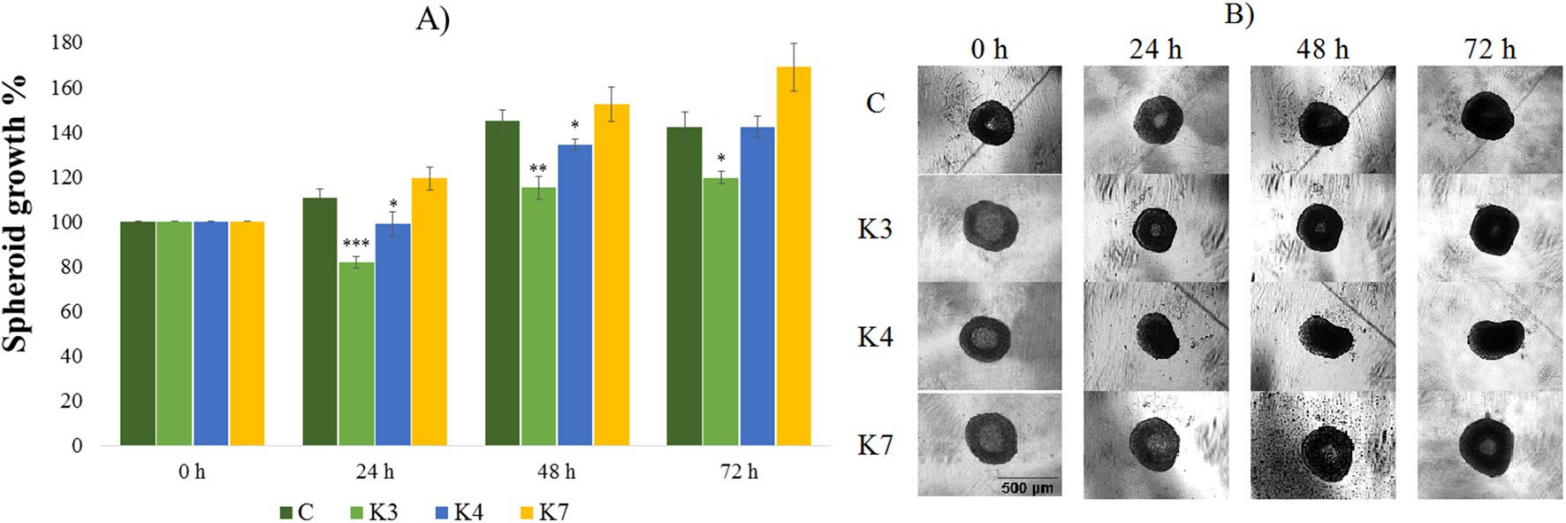
The impact of **K3**, **K4**, and **K7** (1 µM) on sensitive MCF-7 spheroids formation after 24, 48, and 72 h exposure (**A**). Representative images of sensitive MCF-7 spheroids at 0–72 h of experiment (**B**), scale bar = 500 µm. The control spheroids (**C**) were treated with 1% V/V DMSO. After incubation, the spheroid areas were determined and the spheroid growth was calculated (**A**, relative to control). Data are expressed as the average of spheroid growth (%) of nine repetitions with the corresponding standard error of the mean. The statistical differences between the control spheroids and treated spheroids were calculated by using a two-compound t-test at the level *p < 0.05, **p < 0.01, ***p < 0.001.

**Figure 6:**
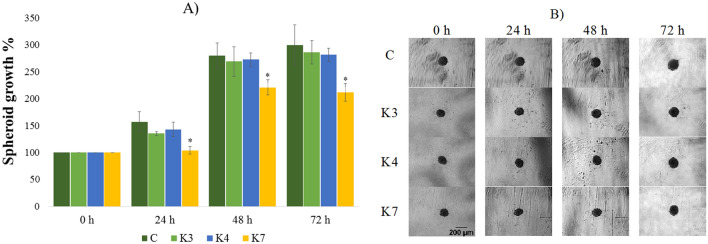
The impact of **K3**, **K4**, **K7** compounds (1 µM) on resistant MCF-7/PAX spheroids formation after 24, 48, and 72 h exposure (**A**). Representative images of resistant MCF-7/PAX spheroids at 0–72 h of experiment (**B**), scale bar = 200 µm. The control spheroids (**C**) were treated with 1% V/V DMSO. After incubation, the spheroid areas were determined and the spheroid growth was calculated (**A**, relative to control). Data are expressed as the average of spheroid growth (%) of nine repetitions with the corresponding standard error of the mean. The statistical differences between the control spheroids and treated spheroids were calculated by using a two-compound t-test at the level *p < 0.05, **p < 0.01, ***p < 0.001.

To monitor the effect of selenoesters on spheroid formation, HOC, HOC/ADR, MCF-7 and MCF-7/PAX cell lines were used. 24 h after HOC cells’ inoculation, the diameter of formed spheroids was measured. The spheroids with diameters above 200 µm are characterized by the presence of a concentration gradient of oxygen, nutrients, and metabolites. The ovarian cancer cells (HOC, HOC/ADR) formed aggregates in which single cells could be recognized (Figs. 3, 4). In contrast, breast cancer cell lines (MCF-7, MCF-7/PAX) (Figs. 5, 6) formed compact spheroids where individual cells could not be distinguished because of close cell–cell interactions. According to this fact, the easier penetration and action of compounds into spheroids of ovarian cancer cells is expected. Upon selenoesters’ addition, the images of spheroids were taken regularly to determine their areas relative to the control spheroids in medium containing the solvent (1% V/V DMSO).

Before the addition of compounds, the spheroid size was considered to be 100% (Figs. 3, 4). The destruction of the spheroids caused by the presence of the tested compounds for 24, 48, and 72 h was determined. The inhibition of the spheroid growth and the decrease of cell number in affected spheroids compared to the control ones in the corresponding time was observed. Selenoesters significantly affected the spheroid formation. The 24 h exposure of HOC spheroid to **K7** inhibited the growth by more than 40% (Fig. 3). The same extent of inhibition was observed throughout the 72 h of treatment. The statistically significant inhibition of HOC spheroid growth was caused by all ketone-selenoesters. After 24 h, all the tested selenocompounds inhibited the spheroid growth below 75%. The prolongation of the incubation time of the spheroids with selenoesters did not induce other significant changes in spheroids size and microscopic structure.

The effect of tested compounds on the growth and resistance of HOC/ADR cell line is shown in Fig. 4. At the time zero, the spheroid growth was arbitrarily considered as 100%. The addition of **K7** decreased the spheroid area to 75% compared to the control (p < 0.01). However, the inhibitory effect of **K7** was lower after longer incubation. The most significant inhibition was caused by **K3** that decreased the spheroid growth by 45% (p < 0.001) during the first 24 h of exposure. Despite the significant spheroids’ growth inhibitory effect of **K3**, the cells of the spheroids overcame the effect of the compound and continued in spheroid formation (Fig. 4). HOC/ADR 3D models showed a higher growth rate in comparison to HOC and during 72 h of incubation, the control spheroid areas more than doubled.

3D breast spheroids (sensitive MCF-7 cell line and its sub-line resistant to paclitaxel) were more compact than that formed by the ovarian cell lines (Figs. 5, 6). To form 3D models with the appropriate diameter, more cells were initially seeded (1 × 10^5^ cells/mL) compared to the ovarian cell line (0.5 × 10^5^ cells/mL). Resistant MCF-7/PAX sub-line produced spheroids with smaller diameter (200 µm) in comparison with MCF-7 cells (500 µm), which can be caused by a more compact arrangement of the cells inside the spheroids (Fig. 6). A lower penetration of the compounds into the spheroid core and thus a lower inhibitory effect can be expected in compact spheroids. Indeed, the size of the spheroids formed by sensitive cells was significantly reduced by **K3**, which decreased the spheroid growth during the whole treatment under 80% (Fig. 5). Some changes in the MCF-7 spheroid areas were caused by **K4** and **K7**, but the impact was lower than that of **K3**. On the contrary, the MCF-7/PAX spheroids were highly resistant to the selenoesters. During the first 24 h, **K7** slowed down the spheroid´s growth, but at longer incubation times, the effect of the compound disappeared (Fig. 6). To conclude, the most significant inhibition was found in ovarian spheroids (HOC, HOC/ADR) where some of the compounds caused the reduction of growth under 60%.

### Cytotoxic activity of selected selenoesters against 3D models of ovarian and breast cancer

The ability of selenoesters to affect the formation of spheroids was described in the previous section. Subsequently, we have tested the cytotoxic potential of selenoesters in preformed 72 h old spheroids. Both sensitive and resistant mature breast and ovarian cells spheroids were incubated in the presence of selenoesters in a concentration range 1.6–100 µM. **K3** demonstrated a significant cytotoxic effect on all tested cell lines and sub-lines (Table 7). All the compounds showed strong cytotoxic effect in MCF-7. A safe concentration of selenoesters has been established based on the results of sensitization of drug-resistant 3D models.

**Table 7:** Cytotoxic activity of selenoesters against 3D ovarian (HOC) and breast (MCF-7) cancer cell models and their Adriamycin (HOC/ADR) and paclitaxel (MCF-7/PAX) resistant sub-models.

Cpd	HOC	IC_50_ [µM]		MCF-7/PAX
		HOC/ADR	MCF-7	
K3	1.7 ± 0.1^a^	3.9 ± 0.2^a^	2.5 ± 0.2^a^	4.0 ± 0.1^a^
K4	3.1 ± 0.1^b^	10.5 ± 0.5^b^	2.1 ± 0.0^a^	4.8 ± 0.3^a^
K7	2.8 ± 0.1^b^	10.1 ± 0.5^b^	2.2 ± 0.3^a^	7.5 ± 0.2^b^

Data are expressed as the average inhibitory concentration (IC_50_) of four repetitions with standard error of the mean (SEM). The statistical differences between compounds were calculated by one-way analysis of variance (ANOVA) and Duncan’s post hoc test (p ˂ 0.05) within one cell line, where different letters (a, b) denoted the significances; the different cell lines were evaluated independently on each other.

### Sensitization of resistant ovarian and breast cancer spheroids by selected selenoesters

In the previous section, the cytotoxic activity of selenoesters was determined against several 3D models of sensitive and resistant cell lines. Subsequently, the selenoesters were tested for their potential to sensitize the drug-resistant HOC/ADR and MCF-7/PAX spheroids. Mature 72 h old spheroids were incubated with the non-toxic concentration of selenoesters with Adriamycin or paclitaxel (concentration range 0.6–80 µM) for HOC/ADR or MCF-7/PAX, respectively (Table 8). After another 72 h incubation, the sensitization rate (fold change) was evaluated. A fold change greater than 1 indicated synergistic effect of the cytotostatic drug and the tested compound. The highest sensitization rate of ovarian spheroids (HOC/ADR) was induced by 5 µM **K4**, followed by 5 µM **K7**, and 3 µM **K3**. On the contrary, no modulation activity was achieved in MCF-7/PAX spheroids, where the calculated fold change was around 1 indicating no synergistic effect between paclitaxel and selenoesters.

**Table 8:** Sensitization of resistant 3D ovarian (HOC/ADR) and breast (MCF-7/PAX) cancer spheroids by selected selenoesters.

Cpd	HOC/ADR		MCF-7/PAX	
	Tested concentration [µM]	Fold change	Tested concentration [µM]	Fold change
K3	3	(1.2 ± 0.2)×	3	(0.9 ± 0.0)×
K4	5	(2.3 ± 0.1)×	3	(1.0 ± 0.1)×
K7	5	(1.6 ± 0.1)×	5	(1.1 ± 0.1)×

Data are expressed as the average sensitization rate (fold change) of four repetitions with standard error of the mean (SEM). Fold change was determined as the ratio of Adriamycin/paclitaxel IC_50_ and Adriamycin/paclitaxel + selenoesters IC_50_.

## Discussion

Selenium (Se) is an essential trace element that participates in many physiological processes. As part of selenoproteins (e.g., glutathione peroxidases, thioredoxin reductases), it maintains a redox system that protects cells against oxidation stress^21^. Additionally, Se-proteins may influence cancer progression by the interaction via tumour microenviroment (TME) and activation of the inflammatory pathways^22^. In cancer research, different types of Se-containing compounds have been developed and investigated (e.g., selenoamino acid derivatives, selenides, selenocyanates and Se-containing heterocycles)^14,22^. In our study, eight ketone-selenoesters and seven cyanoselenoesters were evaluated for their anticancer and MDR modulation potential using 2D and 3D cultures.

Cyanoselenoesters caused a lower decrease in viability of both Adriamycin sensitive (HOC) and resistant (HOC/ADR) ovarian adenocarcinoma cells compared to ketone-selenoesters, which were highly toxic. The distinct results were received for breast cancer cells MCF-7 and MCF-7/PAX in the anticancer potential of all the compounds tested was quite consistent. **K1** and **K2** compounds caused the most significant decrease of viability of all tumour cell lines tested. These results agreed with those published in our previous article; where **K1** was among the first three most active compounds against colon adenocarcinoma cells—sensitive Colo 205 and Adriamycin-resistant Colo 320 colon adenocarcinoma, respectively, and against hepatocellular carcinoma cell line HepG2. In the case of **K2**, the highest cytotoxic potential was also observed in human hepatocellular carcinoma cell line HepG2 and in skin melanoma cell line B16^17^.

Similarly, the ketone-selenoesters (except for **K4**) were more potent inhibitors of the non-cancerous embryonic kidney cell line HEK293 than the cyanoselenoesters. **K1–3**, **K5**, **K7–8** showed the most significant toxicity, what is in correlation with our previous results^17^. **K1** and **K2** achieved the lowest IC_50_ values for all tested cell lines. We assume that the thionyl ring present in **K1** increases the anticancer potential, as well as the fluorine substituent on the **K2** ring. As described in another study, the ketone-containing selenoesters showed the most significant cytotoxic potential against Colo 205/Colo 320 (sensitive and Adriamycin-resistant colon adenocarcinoma), MCF-7 (sensitive breast adenocarcinoma), KCR (Adriamycin-resistant breast sub-line)^23,24^. From the point of view of collateral sensitivity^25^, **K8** and **N1** provided a slight selective effect against MCF-7/PAX and **N2–3** in HOC/ADR. By comparing cytotoxicity with that studied on non-cancerous HEK293 cell line, **N2–3** demonstrated a moderate selectivity against both paclitaxel-sensitive and resistant MCF-7 cells, **N7** against sensitive MCF-7. In our previous study, all the cyanoselenoesters showed strong cancer selectivity for non-cancerous MRC-5 human embryonal lung fibroblasts in comparison with Colo 205, Colo 320, HepG2, HeLa, and B16 cell lines^17^.

During long-term treatment with chemotherapeutics, some tumours can activate mechanisms by which cells become resistant to multiple drugs, which is known as multidrug resistance (MDR). Despite significant advances in medicine, chemotherapy remains one of the few cancer treatment options. Well-designed MDR modulators that synergistically enhance the potential of anticancer agents represent a progressive approach to overcome this phenomenon. In recent years, ABC inhibitors showed promising results not only in the in vitro and in vivo experimental conditions, but also in clinical trials^26–28^. Searching for new ABC modulators with higher selectivity is more than desirable. Regarding this problem, we have tested the potential of 15 selenocompounds to sensitize HOC/ADR. The most significant sensitization was caused by **K7** (IC_10_ = 1.8 µM), thus the addition of compound decreased the IC_50_ of Adriamycin almost sixfold. No statistically significant differences were found between IC_50_ of Adriamycin alone or in a combination with most of the tested selenocompounds, except for **K5**, **N1** and **N6**. The three compounds provided an antagonistic effect with cytostatic agent, caused higher Adriamycin concentration needed for the inhibition of cell viability. Therefore, we speculate that these compounds could activate MDR transport pumps. Our previous results from the checkerboard combination assay performed on MDR Colo 320 cells proved the existence of synergistic dose-dependent interactions between **K1**, **K3**, **K4**, **K6**, **K8**, **N2–4**, **N7** and Adriamycin^17^. These results agreed with our new data in this study, which indicate a slight synergistic effect observed on HOC/ADR cells. **K7** displayed also the most significant sensitization of MCF-7/PAX cells, when it lowered the IC_50_ of paclitaxel approximately twofold. A slight paclitaxel sensitization was caused also by **K3** and **K4**. Regardless **K7** acts either as a competitive or non-competitive inhibitor, the 4-*tert*butylphenyl ring probably facilitates the interaction with the drug-binding or nucleotide-binding domain of the pump^29^. Despite similar results observed on these drug-resistant cell lines, the tumours differ in gene expression and response to therapy, therefore, each cell line is unique from the MDR perspective^30^.

We demonstrated the potential mechanism of action by which compounds **K3**, **K4**, and **K7** provided anticancer and MDR modulation activity. Selected ketone-selenoesters (**K3**, **K4** and **K7**) were investigated for their induction of, oxidation stress potential, direct inhibition of P-gp, and modulation of ABC transporter expression. Adriamycin is a well-known inductor of apoptosis, but also induces other mechanisms of cell death such as autophagy, necroptosis, ferroptosis, pyroptosis, and others^31^. We hypothesize that the presence of a potential MDR inhibitor (**K3**, **K4** and **K7**) in P-gp overexpressing HOC/ADR cells will increase apoptotic events due to inhibition of efflux of Adriamycin. In another study, the combination of Adriamycin and verapamil caused synergistic effect in Adriamycin-resistant human leukaemia cell line K562/ADR manifested as a higher apoptosis rate^32^.

We tested also the ability of selenoesters to induce generation of reactive oxygen species (ROS) on HOC cell line. A higher dose of DMSO (5% V/V), a well-known activator of oxidation stress^33^, caused a massive increase of ROS positive cells. All tested compounds affected ROS production in some negative extent. **K3** and **K7** increased the ROS production more significantly than **K4**. We concluded that **K3** and **K7** showed high cytotoxic potential against almost all cell lines due to increased ROS generation. For normal cells, a moderate level of ROS represents biomolecules essential for proliferation and some of the defence mechanisms. In cancer cells, the ROS level increases antioxidant processes. However, when the redox level exceeds a tolerable limit, oxidation stress triggers cell death through apoptosis, necrosis, and autophagy^34^. Our results proved that ketone-selenoesters can significantly increase intracellular concentration of ROS. However, the mechanism of cell death remains to be elucidated. In fact, selenium compounds affected ROS levels that led to enhanced cytotoxicity and subsequently activation of cell death^35,36^. As cancer cells have generally higher ROS levels than normal cells, increasing slightly or moderately ROS levels could lead to a selective exceeding of the threshold limit in cancer cells in respect to non-cancer ones. This strategy has been applied in both selenocompounds^37^ and tellurocompounds^38^. This effect converts selenium in an interesting element, as can act as antioxidant^35,39^ or as a pro-oxidant^38^, depending on the compound and the environment^37^.

The increased number of patients with resistant tumours justifies the search for new effective MDR modulators. The etiology of MDR in cancer is highly complex. There are many factors that contribute to the development of MDR, such as changes in induction of expression of apoptosis genes of the Bcl-2 family, increased activity of detoxification enzymes or growth factors. Another very common factor is the overexpression of genes encoding ABC superfamily efflux pumps, especially of P-glycoprotein^2^. The modulators can interact directly with the drug binding site as competitive inhibitors, inhibit ATP binding to the ATP binding site as non-competitive inhibitors, or modulate the active P-gp conformation by interaction with an allosteric residue as a non-competitive inhibitor^40^. In previous studies, the P-gp modulating activity of several selenoesters was evaluated^16,23,24,41^. We noticed that **K3**, **K4**, and **K7** were able to inhibit verapamil-stimulated P-gp ATPase activity in a dose-dependent manner. The most effective inhibition was obtained by **K3** - its highest concentrations inhibited P-gp ATPase activity more strongly than sodium orthovanadate (non-competitive P-gp inhibitor). Our previous results showed that **K3** and **K7** effectively inhibited rhodamine 123 efflux causing its intracellular accumulation^42^. **K7** with a 4-*tert*butylphenyl ring showed an activity similar to that of **K3** with 4-bromophenyl moieties. The mechanism of P-gp inhibition (competitive or non-competitive mode) was revealed, after the incubation of compounds with verapamil-stimulated P-gp membranes. Based on comparison with the preceding data without the addition of verapamil, it is clear that **K7** acted as a P-gp inhibitor, decreasing the ATP consumption and **K4** showed almost no impact on ATPase activity. In contrast, **K3** alone stimulated P-gp activity resulting in higher ATP consumption comparable to verapamil. This indicates that **K3** as the P-gp substrate competitively inhibits efflux of another substrate. An opposite situation was observed in case of **K7** which in both approaches acted as a P-gp inhibitor, thus this compound could be considered as a non-competitive P-gp inhibitor. Regarding **K4**, our results proved that P-gp activity was inhibited in a dose-dependent manner. On the other hand, one from the tested concentration of **K4** alone did not affect P-gp activity. That is why we speculate that the effect of the compound will be concentration dependent and higher doses of **K4** will activate the P-gp efflux as the substrate (competitive inhibitor)^42^. Tariquidar provided similar results; it acted as a BCRP substrate at lower concentrations in contrast to higher concentrations where it caused BCRP competitive inhibition^43^.

Another approach to overcome MDR is associated with modulation of expression of genes encoding ABC proteins. We previously described that the drug resistant phenotype of the HOC/ADR cell line is related to overexpression of ABCB1 (P-gp) and ABCC1 (MRP1)^18^. With regarding to MCF-7/PAX cells, expression of genes encoding ABCB1 and ABCG2 was significantly increased compared to the sensitive cell line (MCF-7)^20^. Our group has recently published downregulation of the *ABCB1* gene by flavonolignans (e.g., silybin B, anhydrosilychristin and isosilychristin)^18,44^. Many transcription factors such as NF-κB (Nuclear Factor Kappa B); β-catenin; AP-1 (Activator protein 1) were found to be involved in P-gp downregulation via cellular signalling pathways^45^. Interestingly, P-gp positive gastric cancer was modulated by tamoxifen through the PK3K/Akt signalling pathway^46^. Additionally, dasatinib was able to alter *ABCB1* expression level by inhibiting the ERK (Extracellular signal-related kinase) pathway activation in Adriamycin-resistant breast cancer cells (MCF-7/ADR)^47^.

Unfortunately, many candidate drugs that provide promising results in vitro appear to be ineffective in vivo due to the high complexity and heterogeneity of various tumours. One of the reasons is that the in vitro assays are usually based only on adherent cultures and do not respect the complex microenvironment of solid tumours. A spheroid model should reliably represent the size-induced microenvironmental changes like cellular heterogeneity, hypoxic gradients, and spatial distribution of necrotic and proliferating cells^48,49^. Therefore, we have tested the anticancer and MDR modulation potential of selected selenoesters on 3D ovarian and breast cancer cell models. We have applied two main approaches: the impact of compounds on spheroid formation; cytotoxic and sensitization activity against mature ovarian and breast cancer 3D models, both sensitive and resistant to cytostatic agents. Both resistant and sensitive ovarian and breast cancer cell lines readily formed spheroids. As we expected, the penetration of the tested compounds was much easier into the ovarian aggregates, thus the formation of sensitive ovarian spheroids was highly affected by all tested compounds. **K3** and **K7** were effective against HOC/ADR spheroids growth predominantly in the first 24 h after the addition of the compounds. **K3** showed the most significant cytotoxic activity on cells of both ovarian 3D models. The IC_50_ values of **K3** for the 2D and 3D HOC cell cultures were almost the same, which would favour this compound as a promising anticancer agent. However, **K3** being a P-gp substrate (competitive inhibitor) carries a higher risk of developing MDR. Therefore, co-treatment combining **K3** with another anticancer agent would represent a better approach^17,50^. On the other hand, both sensitive and resistant MCF-7 breast cancer cells formed compact spheroids and selenoesters showed a slight effect on spheroid formation except for **K3**, which affected spheroids during the first 48 h of incubation. Compared to MCF-7, mature MCF-7/PAX spheroids displayed a higher degree of resistance to the cytotoxic effect of selenoesters and MDR modulation due to lower drug penetration. Ruiz et al. compared cytotoxic effects of organoruthenium complexes on 2D and 3D models of bone, lung and breast cancer. In MCF-7 spheroids, Ru-complex 1 achieved almost fourfold higher IC_50_ comparing to 2D models, and Ru-complex 2 almost 18-fold^51^.

Due to their significant anticancer and MDR modulating activity, the application of ketone-selenoesters may be considered as a promising approach to treat drug-resistant tumours. Besides, **K7** as the non-competitive P-gp inhibitor meets the criteria for successful clinical use of P-gp modulators. As future plans, a targeted delivery system can provide selectivity for cancer tissue. Moreover, a retarded controlled release of the cytostatic agent may decrease the systemic toxicity and improve drug distribution and circulation time^52^. The potential of selenocompounds is also underlined by the reported use of biogenic selenium nanoparticles for prostate cancer therapy with higher specificity both in vitro and in vivo experiments^53^.

## Methods

### Topical compounds

Eight ketone- and seven cyano-selenoesters were synthesized and characterized according to the previously described procedure^17^. Before biological testing, the compounds were dissolved in dimethyl sulfoxide (DMSO) and the stock solution with 10 mM concentration was prepared.

### Analytical standards and chemicals

From Sigma Aldrich (Saint Louis, Missouri, United States) were purchased the following standards or chemicals: doxorubicin hydrochloride (purchased under ‘Adriamycin’ trade name), 100× antibiotic antimycotic solution, Dulbecco’s Modified Eagle’s medium—high glucose (or DMEM), Eagle’s minimum essential medium (EMEM), dimethylsulfoxide (DMSO), paclitaxel, l-glutamine solution, fetal bovine se-rum (FBS), trypsin-2,2′,2″,2‴-(ethane-1,2-diyldinitrilo)tetraacetic acid (EDTA) solution and resazurin sodium salt. From other providers, the following items were also used: CellTiter-Glo^®^ 3D Cell Viability Assay and P-gp-Glo Assay system (Promega, Madison, Wisconsin, United States); Muse™ Annexin V & Dead Cell Assay and Muse^®^ Oxidative Stress Kit (Luminex Corporation, Austin, Texas, United States); Quant-iT RiboGreen RNA Assay Kit (Invitrogen, Waltham, Massachussets, United States); RevertAid First Strand Synthesis cDNA Kit (MBI Fermentas, Vilnius, Lithuania); SeaKem LE Agarose (Lonza Bioscence, Walkersville, Maryland, United States); Trizol Reagent (Thermo Fisher Scientific, Waltham, Massachussets, United States); 20× TaqMan Gene Expression Assay (Life Technologies), 5× HotFIREPol Probe qPCR Mix Plus (Solis Bio-dyne, Tartu, Estonia).

### Cytotoxic activity of selenoesters against several cancer and non-cancerous cell lines

The cytotoxic potential of selenoesters was tested on several cancer cell lines—HOC (human parental ovarian adenocarcinoma, HOC, A2780, Sigma-Aldrich), HOC/ADR (sub-line resistant to Adriamycin, A2780/ADR, Sigma-Aldrich), MCF-7 (human breast adenocarcinoma, ATCC^®^, HTB-22TM), MCF-7/PAX (resistant sub-line established by gradual adaptation of parental cells to increased concentration of paclitaxel; kindly provided by Division of Cell and Molecular Biology, Third Faculty of Medicine, Charles University, Czech Republic) and on one non-cancerous cell line—HEK293 (human embryonic kidney cell line, Leibniz Institute, DSMZ-German Collection of Microorganisms and Cell Cultures GmbH). All mentioned cell lines were authenticated using short tandem repeat (STR) profiling. Cell lines were cultivated in DMEM medium (10% FBS, 1× antibiotic antimycotic solution), except for HEK293 cell line which was cultivated without antibiotics. In case of the resistant cell lines, the medium was supplemented with a required concentration of cytostatics to maintain resistance (HOC/ADR—100 nM Adriamycin; MCF-7/PAX—300 nM paclitaxel). All cells were cultivated in a CO_2_ incubator (5% CO_2_, 37 °C, Thermo Fisher Scientific).

For the experiment, the cells at passage number 5–20 were seeded at a 1 × 10^5^ cells/mL concentration into 96-well plates to 100 µL final volume^44^. The final concentration range of the compounds was 0.63–20 µM. After an incubation of 72 h, the plates were washed 1× with PBS and resazurin solution (0.03 mg/mL in 1× PBS) was added. After 1 h of incubation, the fluorescence signal was recorded (ex./em. 560/590 nm).

The selectivity index (SI) was determined as the ratio of IC_50_ value for the non-cancerous cells to IC_50_ for the cancer cell line. For resistant cell lines, the collateral sensitivity (CS) was calculated as the ratio of IC_50_ value in sensitive tumour cells to IC_50_ in the resistant cancer cell line. In both cases, the selectivity is considered as: (1) strongly selective when SI or CS value is above 6, (2) moderately selective when SI or CS is between 3 and 6, (3) slightly selective when SI or CS is between 1 and 3 and (4) non-selective when SI or CS is less than 1^23^.

### Sensitization of MDR cell lines by selenoesters

To prove the sensitization potential of compounds, both multidrug resistant cell lines (HOC/ADR and MCF-7/PAX) were seeded at the concentration of 1 × 10^5^ cells/mL into 96-well plates and incubated 24 h. After that, the plates were washed 1× with PBS and fresh DMEM medium supplemented with tested compounds at final concentration corresponding their IC_10_ value was added, except for positive control wells. Subsequently, Adriamycin (concentration range 0.1–20 µM) or paclitaxel (concentration range 0.1–10 µM) was added to the appropriate cell line for 72 h. After incubation, the cells were washed 1× with PBS and the resazurin solution (0.03 mg/mL) was added to evaluate the cell viability as described above. IC_50_ values of cytostatics were calculated for both the control and compounds; a fold change was counted up. When this fold change is higher than 1 indicates a synergism between the tested cytostatic agent and the compound, while a fold change lower than 1 means an antagonism.

$$\text{Fold change}=\frac{{\text{IC}}_{50}\text{ value of cytostatic treated cells only}}{{\text{IC}}_{50}\text{ value of cytostatic treated cells in addition of the compound}}$$

### Oxidation stress potential of selected selenoesters

To determine the oxidation stress potential of selected selenoesters, HOC were seeded into 24-well plates at a density 2 × 10^5^ cells/mL for 24 h according to the above mentioned procedure. Afterwards, the plates were washed 1 ×with PBS and fresh DMEM medium containing compounds **K3**, **K4**, **K7** (final concentration—1 µM) was added for 24 h, except for the positive and negative control. Positive control represented non-treated cells in DMEM medium with 1% (V/V) of DMSO and negative control with 5% (V/V) DMSO as the oxidation stress inductor. After incubation, the cells were washed 1× with PBS and released from the surface by using a trypsin–EDTA solution. After trypsin inhibition by medium addition, the cell suspensions were centrifuged (100×*g*, 5 min, 25 °C), supernatants were removed, and cell pellets resuspended in 1× assay buffer. All reagents were prepared immediately before use. Afterwards, 10 µL of the cell suspension were mixed with 190 µL of working solution (Muse Oxidative stress reagent) and stained for 30 min at 37 °C. Subsequently, the compounds were analysed by Guava Muse Cell Analyzer. Oxidation stress detection was performed following the instructions provided by the manufacturer.

### P-glycoprotein modulation by selenoesters

The modulation of P-glycoprotein ATPase activity was performed according to the manufacturer’s guidelines^54^ and previously published work^55^. Briefly, a human recombinant P-gp membrane fraction (25 µg per reaction) was mixed with assay buffer (basal control); verapamil (P-gp activator, 200 µM); Na_3_VO_4_ (P-gp inhibitor, 100 µM); ATP standards (0.375–3 mM standard curve) and compounds **K3**, **K4**, **K7** (the concentration range of 12.5–100 µM). To examine the inhibitory potential, 10 µL of verapamil was added to the reaction with the compounds. The reduction of verapamil-stimulated ATPase activity by selenoesters can indicate them as P-gp ATPase inhibitors. The reaction was initiated with 10 µL of MgATP (5 mM) addition to the final 50 µL volume. After 1 h incubation at 37 °C, the ATPase reaction was stopped with 50 µL of ATP detection reagent and subsequently the plate was incubated for 20 min at room temperature. Thanks to the luciferase reaction, it was possible to detect the remaining non-metabolized ATP as the luminescent signal. By using ATP standards and linear regression, the relative luminescent values (RLU) were converted to ATP concentration per reaction; and the amount of consumed ATP by the compounds was calculated.

### Effect of selenoesters on expression of genes encoding ABC transporter

To determine the impact of selected compounds on the expression of genes encoding ABC transporter, HOC/ADR and MCF-7/PAX cells were seeded for 24 h into 5 cm Petri dishes with DMEM medium at the cell density 1 × 10^5^ cells/mL. Afterwards, the cells were washed 1× with PBS and new fresh DMEM supplemented with **K3**, **K4**, and **K7** (in concentration corresponding to their IC_10_ value) alone or combined with cytostatics (Adriamycin or paclitaxel in concentration corresponding to their IC_25_ value) was added. In the experiment, positive and negative control was also included (positive—untreated cells, negative—cells treated only with cytostatics). After 48 h exposure, the cells were washed once with PBS and released from the surface by using the above-mentioned trypsin–EDTA procedure. The enzyme reaction was stopped by medium addition and the cells were centrifuged (3200×*g*; 10 min, 4 °C). The supernatants were removed and before the next centrifugation, the pellets were resuspended in 1.5 mL of cooled PBS (5400×*g*; 10 min, 4 °C). After the second washing step, the cells were centrifuged another time (10,000×*g*; 3 min, 4 °C). Subsequently, 1 mL of Trizol (Invitrogen) was added to the pellets and the compounds were stored − 80 °C.

The RNA concentration of selenoester treated samples was determined using QuantiT RiboGreen RNA Assay Kit and Infinite M200 plate reader (Tecan, Männedor, Switzerland). The cDNA was synthesized from 0.5 µg of total RNA by RevertAid First Strand Synthesis cDNA Kit (MBI Fermentas, Vilnius, Lithuania). To determine the quality of cDNA, the ubiquitin C gene fragment was amplified. The experiment was performed in 384-well block by using ViiA7 Real-Time PCR System (Life Technologies, Camarillo, California, United States). A master mix contained 0.25 µL of 20× TaqMan Gene Expression Assay, 1 µL of 5× HotFIREPol Probe qPCR Mix Plus, 1.75 µL of RNase free water and 2 µL of 8× diluted cDNA. The final reaction volume was 5 µL. The PCR parameters were as follows: initial hold (50 °C for 2 min), denaturation (95 °C for 10 min), followed by 45 cycles (denaturation at 95 °C for 15 s, annealing at 60 °C for 1 min). After each cycle, the fluorescent signal was determined. For each compound, duplicates were analysed and those with a standard deviation higher than 0.5 Ct were re-analysed. A quantitate real-time PCR was followed by MIQE guidelines^56^. To compare the relative transcript levels of genes, the software REST 2009 (Qiagen, Hilden, Germany) was used.

### The impact of selected selenoesters on 3D spheroids formation

Human ovarian and breast cancer cells (parental and resistant) were seeded into U bottom 96-well plates (VWR, Radnor, Pennsylvania, United States) coated with 0.8% SeaKem LE Agarose at a cell density 0.5 × 10^5^ cells/mL (HOC, HOC/ADR) and 1 × 10^5^ cell/mL (MCF-7, MCF-7/PAX) in 100 µL of medium with appropriate cytostatic agent. After 24 h, the plates were enriched with 100 µL of medium with tested compounds **K3**, **K4**, **K7** at the final concentration of 1 µM, except for control cells (100 µL medium with 1% V/V DMSO). Spheroids were recorded 0; 24; 48 and 72 h after exposure to tested compounds by using the light microscope Axio Vert. A1 (Zeiss, Jena, Germany) with photo documentation equipment, Axiocam ICC 1 and Axio Vision 4.8 software (Zeiss), and the spheroid areas were determined by using ImageJ (National Institute of Health, Bethesda, Maryland, United States). Subsequently, a spheroid growth was calculated according to equation:

$$\text{Spheroid area }\left(\%\right)=\frac{\text{SAS at t}}{\text{SAC at t}} \times 100$$

where SAS represents the area of spheroids treated with the compound; SAC is the control spheroid area; and the exposure time (t = 0; 24; 48; 72 h).

### The effect of selenoesters on size and compactness of spheroids of ovarian and breast cancer

Human ovarian and breast cancer cells (HOC, MCF-7, parental and resistant) were seeded in ultra-low attachment 96-well plates (Sigma-Aldrich) at a cell density of 0.5 × 10^5^ cells/mL. After 3 days of spheroid formation, plates were carefully washed once with PBS and fresh DMEM medium (99 µL) was added. Using a binary compound dilution, the concentration range of the **K3**, **K4**, **K7** compounds was prepared in new 96-well plates (0.16–10 mM). After 1 µL compound transfer, the final concentration range of compounds was 1.6–100 µM. The required volume of DMSO (1% V/V) was added to the positive control. After 72 h of incubation, 100 µL of CellTiter-Glo 3D Re-agent (Promega) was added to each well. Spheroid plates were incubated for 25 min at room temperature. Subsequently, the luminescent signal was recorded by detector (SpectraMax i3x Multi-Mode Microplate reader with MiniMax Imaging Cytometer, Molecular Devices).

### Sensitization of resistant 3D ovarian and breast cancer spheroids by selected selenoesters

To determine the sensitization potential of compounds, HOC/ADR and MCF-7/PAX were seeded into the ultra-low attachment 96-well plates (Sigma-Aldrich) at a cell density 0.5 × 10^5^ cells/mL. After 3 days of spheroid formation, spheroids were carefully washed once with PBS and fresh DMEM medium (99 µL) containing the appropriate concentration of compounds **K3**, **K4**, **K7** was added. Using a binary serial compound dilution, the concentration range of both cytostatics was prepared in the new 96-well plates (0.06–8 mM). After 1 µL transfer of cytostatics into the corresponding cell line, the final concentration range were 0.6–80 µM. The required volume of DMSO (1% V/V) was added to the positive control. After an incubation of 72 h, 100 µL of CellTiter-Glo 3D Reagent (Promega) was added to each well. Plates with spheroids were incubated for 25 min at room temperature. Afterwards, the luminescent signal was recorded.

### Statistical analysis and data processing

For each experiment, an appropriate number of repetitions (n) was performed. The relative compound activity (RA) within the individual tests was determined as a percentage according to an equation:

$$\text{RA}\left(\%\right)=100*\frac{\text{slope of compound}-\text{average slope of NC}}{\text{average slope of PC}-\text{average slope of NC}}$$

Being NC the negative control and PC, the positive control. To determine IC_50_ values by using non-linear regression, GraphPad Prism 7 software (GraphPad Software, San Diego, California, United States) was used:

$${\text{Y}} = \frac{{{\text{Bottom}} + \left( {{\text{Top}} - {\text{Bottom}}} \right)}}{{1 + 10^{ \wedge } \left( {\left( {{\text{LogIC}} - {\text{X}}} \right)*{\text{HillSlope}}} \right)}}$$

The data averages were calculated and presented with the standard error of the mean (SEM). By using Excel t-test function, the statistical significance was determined. Data were analysed with one-way analysis of variance (ANOVA, Statistica 13, Tibco Software Inc., Tulsa, Oklahoma, United States) and Duncan’s post hoc test. The differences between the groups were considered as statistically significant when p < 0.05.

## Supplementary Information

Supplementary Information.
