# Supplementary material for: Ketone-selenoesters as potential anticancer and multidrug resistance modulation agents in 2D and 3D ovarian and breast cancer in vitro models
Source: Sci Rep. 2022 Apr 21;12:6548. doi: 10.1038/s41598-022-10311-y (PMC9023544; doi:10.1038/s41598-022-10311-y)
Supplement: Supplementary file 1 — Supplementary Information. [file 41598_2022_10311_MOESM1_ESM.docx]

**Supplementary material**

**Table 1.** Effect of selenoesters on the expression of genes of the ABC superfamily in Adriamycin-resistant ovarian carcinoma cell line (HOC/ADR). Expression of ABC genes in HOC/ADR cells treated with selenoesters in concentration of IC_10_ (**K3** = 2.0 µM, **K4** = 2.9 µM, **K7** = 1.8 µM) either alone or in the combination with IC_25_ of Adriamycin (1.25 µM)) was compared to untreated control (in the presence of Adriamycin without compound addition).

|  |  |  |  | **K3** | | **K4** | | **K7** | | **K3+A** | | **K4+A** | | **K7+A** | |
| --- | --- | --- | --- | --- | --- | --- | --- | --- | --- | --- | --- | --- | --- | --- | --- |
| **Gene**  **Symbol** | **Assay ID** | **Genbank Accession No.** | ***P**** | **Expression**  **Difference** | **Treated**  **vs. Untr.** | **Expression**  **Difference** | **Treated**  **vs. Untr.** | **Expression**  **Difference** | **Treated**  **vs. Untr.** | **Expression**  **Difference** | **Treated**  **vs. Untr.** | **Expression**  **Difference** | **Treated**  **vs. Untr.** | **Expression**  **Difference** | **Treated**  **vs. Untr.** |
| PPIA | Hs99999904_m1 | NM_021130.3 |  | 1.000 |  | 1.000 |  | 1.000 |  |  | 1.000 |  | 1.000 |  | 1.000 |
| ABCA1 | Hs00194045_m1 | NM_005502.3 | 0.000 | 0.968 | - | 1.347 | UP | 1.104 | UP | 3.517 | UP | 3.845 | UP | 2.789 | UP |
| ABCA2 | Hs00242232_m1 | NM_212533.2 | 0.000 | 0.997 | - | 0.948 | - | 1.775 | UP | 3.312 | UP | 3.047 | UP | 1.731 | UP |
| ABCA3 | Hs00184543_m1 | NM­_001089.2 | 0.000 | 0.838 | DOWN | 0.832 | DOWN | 1.018 | UP | 1.111 | UP | 1.174 | UP | 0.777 | DOWN |
| ABCA5 | Hs00363322_m1 | NM_172232.2 | 0.000 | 2.693 | UP | 2.334 | UP | 1.970 | UP | 4.522 | UP | 4.443 | UP | 5.333 | UP |
| ABCA7 | Hs00185303_m1 | NM_019112.3 | 0.000 | 0.322 | DOWN | 0.359 | DOWN | 0.341 | DOWN | 0.513 | DOWN | 0.505 | DOWN | 0.477 | DOWN |
| **ABCB1** | **Hs00184491_m1** | **NM_000927.4** | **0.000** | **3.825** | **UP** | **3.696** | **UP** | **5.009** | **UP** | **8.234** | **UP** | **8.623** | **UP** | **6.798** | **UP** |
| ABCB2 | Hs00388677_m1 | NM_000593.5 | 0.000 | 0.315 | DOWN | 0.435 | DOWN | 0.591 | DOWN | 4.074 | UP | 4.517 | UP | 3.613 | UP |
| ABCB3 | Hs00241060 | NM_018833.2 | 0.000 | 4.904 | UP | 4.568 | UP | 3.406 | UP | 3.808 | UP | 3.361 | UP | 5.309 | UP |
| ABCB6 | Hs00180568_m1 | NM_005689.2 | 0.000 | 4.860 | UP | 5.132 | UP | 4.646 | UP | 6.713 | UP | 6.391 | UP | 6.143 | UP |
| ABCB7 | Hs00188776_m1 | NM_004299.3 | 0.000 | 6.3444 | UP | 7.250 | UP | 7.145 | UP | 8.776 | UP | 7.695 | UP | 6.353 | UP |
| ABCB8 | Hs00185159_m1 | NM_007188.3 | 0.000 | 2.659 | UP | 3.526 | UP | 3.581 | UP | 4.321 | UP | 4.521 | UP | 3.569 | UP |
| ABCB9 | Hs00608640_m1 | NM_203444.2 | 0.000 | 4.156 | UP | 4.981 | UP | 4.530 | UP | 5.895 | UP | 6.512 | UP | 5.556 | UP |
| ABCB10 | Hs00429240_m1 | NM_012089.2 | 0.000 | 7.275 | UP | 8.165 | UP | 7.288 | UP | 7.093 | UP | 6.491 | UP | 6.094 | UP |
| **ABCC1** | **Hs00219905_m1** | **NM_004996.3** | **0.000** | **6.415** | **UP** | **8.545** | **UP** | **7.835** | **UP** | **11.68** | **UP** | **12.32** | **UP** | **12.84** | **UP** |
| ABCC2 | Hs00166123_m1 | NM_000392.3 | 0.000 | 2.907 | UP | 3.272 | UP | 3.865 | UP | 5.962 | UP | 6.111 | UP | 6.842 | UP |
| ABCC5 | Hs00981089_m1 | NM_005688.2 | 0.000 | 2.301 | UP | 2.336 | UP | 3.327 | UP | 4.509 | UP | 4.879 | UP | 2.998 | UP |
| ABCC10 | Hs00675716_m1 | NM_033450.2 | 0.000 | 1.562 | UP | 1.545 | UP | 1.899 | UP | 3.411 | UP | 3.418 | UP | 2.443 | UP |
| ABCD1 | Hs00163610_m1 | NM_000033.3 | 0.000 | 0.665 | DOWN | 0.796 | DOWN | 0.898 | DOWN | 2.888 | UP | 3.059 | UP | 1.507 | UP |
| ABCD2 | Hs00193054_m1 | NM_005164.3 | 0.000 | 1.845 | UP | 4.885 | UP | 0.638 | DOWN | 8.898 | UP | 4.847 | UP | 3.491 | UP |
| ABCD3 | Hs00161065_m1 | NM_002858.3 | 0.000 | 2.960 | UP | 3.302 | UP | 4.007 | UP | 5.575 | UP | 5.155 | UP | 4.019 | UP |
| ABCD4 | Hs00245534_m1 | NM_005050.3 | 0.000 | 2.760 | UP | 2.681 | UP | 3.541 | UP | 5.517 | UP | 4.931 | UP | 3.866 | UP |
| ABCE1 | Hs01009190_m1 | NM_001040876.1 | 0.000 | 7.298 | UP | 6.945 | UP | 10.154 | UP | 7.970 | UP | 8.757 | UP | 6.397 | UP |
| ABCF1 | Hs00153703_m1 | NM_001090.2 | 0.000 | 1.638 | UP | 1.998 | UP | 2.212 | UP | 2.991 | UP | 2.083 | UP | 2.601 | UP |
| ABCF2 | Hs00606493_m1 | NM_005692.4 | 0.000 | 2.868 | UP | 3.096 | UP | 4.029 | UP | 4.491 | UP | 5.470 | UP | 3.360 | UP |
| ABCF3 | Hs00217977_m1 | NM_018358.2 | 0.000 | 2.163 | UP | 2.262 | UP | 3.001 | UP | 4.057 | UP | 4.502 | UP | 3.033 | UP |
| ABCG1 | Hs00245154_m1 | NM_207629.1 | 0.000 | 0.932 | - | 1.213 | UP | 1.059 | - | 3.361 | UP | 3.527 | UP | 2.717 | UP |
| **ABCG2** | **Hs00184979_m1** | **NM_004827.2** | **0.000** | **1.869** | **UP** | **1.302** | **UP** | **1.468** | **UP** | **3.816** | **UP** | **3.165** | **UP** | **2.887** | **UP** |
| ABCG4 | Hs00223446_m1 | NM_001142505.1 | 0.000 | 0.755 | DOWN | 0.776 | DOWN | 1.431 | UP | 6.171 | UP | 7.351 | UP | 5.398 | UP |

Gene expression of ABC transporters were estimated using comercial predesigned Taqman Gene Expression Assays (Life technologies). TaqMan Gene Expression Assays are based on 5´ nuclease chemistry, and each assay contains the primer and probe set for target of interest. Taqman assays IDs and GenBank Accession numbers of reference sequencies identifying their location and amplicon lengths are included in presented. * Relative transcript levels (P-value ) in treated and non-treated cancer cell lines were compared using REST 2009 software.

**Table 2.** Effect of selenoesters either on the expression of the ABC gene superfamily in paclitaxel-resistant breast cancer cell line (MCF-7/PAX). Expression of ABC genes in MCF-7/PAX cells treated with selenoesters at IC_10_ (**K3** = 1.2 µM, **K4** = 1.8 µM, **K7** = 1.3 µM) either alone or in the combination with IC_25_ of paclitaxel (0.75 µM)) was compared to untreated control (in the presence of paclitaxel without compound addition).

|  |  | **K3** | | **K4** | | **K7** | | **K3+P** | | **K4+P** | | **K7+P** | |
| --- | --- | --- | --- | --- | --- | --- | --- | --- | --- | --- | --- | --- | --- |
| **Gene**  **Symbol** | ***P**** | **Expression**  **Difference** | **Treated**  **vs. Untr.** | **Expression**  **Difference** | **Treated**  **vs. Untr.** | **Expression**  **Difference** | **Treated**  **vs. Untr.** | **Expression**  **Difference** | **Treated**  **vs. Untr.** | **Expression**  **Difference** | **Treated**  **vs. Untr.** | **Expression**  **Difference** | **Treated**  **vs. Untr.** |
| PPIA |  | 1.000 |  | 1.000 |  | 1.000 |  |  | 1.000 |  | 1.000 |  | 1.000 |
| ABCA2 | 0.001 | 0.188 | DOWN | 0.282 | DOWN | 0.159 | DOWN | 0.153 | DOWN | 0.472 | DOWN | 0.259 | DOWN |
| ABCA3 | 0.001 | 0.481 | DOWN | 0.766 | DOWN | 0.376 | DOWN | 0.452 | DOWN | 0.955 | - | 0.808 | DOWN |
| ABCA4 | 0.001 | 27.38 | UP | 40.43 | UP | 20.68 | UP | 16.44 | UP | 45.32 | UP | 31.22 | UP |
| ABCA5 | 0.001 | 0.559 | DOWN | 0.789 | DOWN | 0.600 | DOWN | 0.553 | DOWN | 1.259 | UP | 0.882 | DOWN |
| ABCA7 | 0.001 | 0.5100 | DOWN | 0.757 | DOWN | 0.384 | DOWN | 0.415 | DOWN | 1.124 | UP | 0.648 | DOWN |
| ABCA12 | 0.001 | 1.149 | UP | 1.702 | UP | 0.745 | DOWN | 0.716 | DOWN | 1.881 | UP | 1.315 | UP |
| **ABCB1** | **0.001** | **0.401** | **DOWN** | **0.568** | **DOWN** | **0.361** | **DOWN** | **0.315** | **DOWN** | **0.665** | **DOWN** | **0.641** | **DOWN** |
| ABCB2 | 0.001 | 0.354 | DOWN | 0.467 | DOWN | 0.274 | DOWN | 0.290 | DOWN | 0.526 | DOWN | 0.451 | DOWN |
| ABCB3 | 0.001 | 0.235 | DOWN | 0.323 | DOWN | 0.164 | DOWN | 0.203 | DOWN | 0.324 | DOWN | 0.335 | DOWN |
| ABCB6 | 0.001 | 0.340 | DOWN | 0.524 | DOWN | 0.305 | DOWN | 0.316 | DOWN | 0.674 | DOWN | 0.552 | DOWN |
| ABCB7 | 0.001 | 0.143 | DOWN | 0.214 | DOWN | 0.131 | DOWN | 0.134 | DOWN | 0.188 | DOWN | 0.217 | DOWN |
| ABCB8 | 0.001 | 0.488 | DOWN | 0.683 | DOWN | 0.407 | DOWN | 0.463 | DOWN | 0.778 | DOWN | 0.634 | DOWN |
| ABCB9 | 0.001 | 0.422 | DOWN | 0.639 | DOWN | 0.340 | DOWN | 0.433 | DOWN | 0.728 | DOWN | 0.563 | DOWN |
| ABCB10 | 0.001 | 0.922 | DOWN | 1.163 | UP | 0.822 | DOWN | 0.820 | DOWN | 1.395 | UP | 1.422 | UP |
| **ABCC1** | **0.001** | **0.272** | **DOWN** | **0.398** | **DOWN** | **0.238** | **DOWN** | **0.218** | **DOWN** | **0.504** | **DOWN** | **0.374** | **DOWN** |
| ABCC2 | 0.001 | 0.076 | DOWN | 0.127 | DOWN | 0.061 | DOWN | 0.056 | DOWN | 0.114 | DOWN | 0.103 | DOWN |
| ABCC3 | 0.001 | 15.79 | UP | 26.21 | UP | 15.69 | UP | 16.83 | UP | 37.95 | UP | 29.60 | UP |
| ABCC4 | 0.001 | 0.221 | DOWN | 0.316 | DOWN | 0.213 | DOWN | 0.171 | DOWN | 0.321 | DOWN | 0.361 | DOWN |
| ABCC5 | 0.001 | 1.010 | - | 1.378 | UP | 0.933 | DOWN | 0.835 | DOWN | 2.107 | UP | 1.543 | UP |
| ABCC6 | 0.001 | 0.958 | DOWN | 2.041 | UP | 0.424 | DOWN | 0.710 | DOWN | 1.887 | UP | 1.632 | UP |
| ABCC8 | 0.001 | 0.648 | DOWN | 1.590 | UP | 0.651 | DOWN | 0.902 | - | 2.076 | UP | 2.319 | UP |
| ABCC10 | 0.001 | 0.171 | DOWN | 0.265 | DOWN | 0.170 | DOWN | 0.164 | DOWN | 0.353 | DOWN | 0.239 | DOWN |
| ABCD1 | 0.001 | 0.123 | DOWN | 0.179 | DOWN | 0.104 | DOWN | 0.112 | DOWN | 0.168 | DOWN | 0.164 | DOWN |
| ABCD3 | 0.001 | 0.267 | DOWN | 0.365 | DOWN | 0.247 | DOWN | 0.238 | DOWN | 0.359 | DOWN | 0.399 | DOWN |
| ABCD4 | 0.001 | 0.971 | - | 1.288 | UP | 0.872 | DOWN | 0.812 | DOWN | 1.667 | UP | 1.326 | UP |
| ABCE1 | 0.001 | 0.552 | DOWN | 0.795 | DOWN | 0.505 | DOWN | 0.424 | DOWN | 0.701 | DOWN | 0.830 | DOWN |
| ABCF1 | 0.001 | 2.388 | UP | 3.646 | UP | 2.263 | UP | 2.205 | UP | 2.739 | UP | 3.730 | UP |
| ABCF2 | 0.001 | 0.625 | DOWN | 0.942 | - | 0.601 | DOWN | 0.653 | DOWN | 0.599 | DOWN | 0.976 | - |
| ABCF3 | 0.001 | 0.487 | DOWN | 0.753 | DOWN | 0.484 | DOWN | 0.398 | DOWN | 0.422 | DOWN | 0.838 | DOWN |
| ABCG1 | 0.001 | 0.132 | DOWN | 0.325 | DOWN | 0.307 | DOWN | 0.309 | DOWN | 0.293 | DOWN | 0.278 | DOWN |
| **ABCG2** | **0.001** | **0.250** | **DOWN** | **0.378** | **DOWN** | **0.207** | **DOWN** | **0.186** | **DOWN** | **0.279** | **DOWN** | **0.308** | **DOWN** |
| ABCG4 | 0.001 | 0.204 | DOWN | 0.412 | DOWN | 0.188 | DOWN | 0.349 | DOWN | 0.402 | DOWN | 0.477 | DOWN |

Gene expression of ABC transporters were estimated using comercial predesigned Taqman Gene Expression Assays (Life technologies). TaqMan Gene Expression Assays are based on 5´ nuclease chemistry, and each assay contains the primer and probe set for target of interest. Taqman assays IDs and GenBank Accession numbers of reference sequencies identifying their location and amplicon lengths are included in the Supplementary Table I. * Relative transcript levels (P-value ) in treated and non-treated cancer cell lines were compared using REST 2009 software.
